# The polygenic nature of telomere length and the anti-ageing properties of lithium

**DOI:** 10.1038/s41386-018-0289-0

**Published:** 2018-12-18

**Authors:** Fiona Coutts, Alish B. Palmos, Rodrigo R. R. Duarte, Simone de Jong, Cathryn M. Lewis, Danai Dima, Timothy R. Powell

**Affiliations:** 1 Social, Genetic and Developmental Psychiatry Centre, Institute of Psychiatry, Psychology and Neuroscience, King’s College London, London, UK; 2 National Institute for Health Research Biomedical Research Centre for Mental Health, Institute of Psychiatry, Psychology and Neuroscience at the Maudsley Hospital and King’s College London, London, UK; 3 Department of Psychology, School of Arts and Social Sciences, City, University of London, London, UK; 4 Department of Neuroimaging, Institute of Psychiatry, Psychology and Neuroscience, King’s College London, London, UK

**Keywords:** Gene regulation, Genetic markers, Predictive markers, Risk factors

## Abstract

Telomere length is a promising biomarker for age-related disease and a potential anti-ageing drug target. Here, we study the genetic architecture of telomere length and the repositioning potential of lithium as an anti-ageing medication. LD score regression applied to the largest telomere length genome-wide association study to-date, revealed SNP-chip heritability estimates of 7.29%, with polygenic risk scoring capturing 4.4% of the variance in telomere length in an independent cohort (*p* = 6.17 × 10^−5^). Gene-enrichment analysis identified 13 genes associated with telomere length, with the most significant being the leucine rich repeat gene, *LRRC34* (*p* = 3.69 × 10^−18^). In the context of lithium, we confirm that chronic use in a sample of 384 bipolar disorder patients is associated with longer telomeres (*p* = 0.03). As complementary evidence, we studied three orthologs of telomere length regulators in a *Caenorhabditis elegans* model of lithium-induced extended longevity and found all transcripts to be affected post-treatment (*p* < 0.05). Lithium may therefore confer its anti-ageing effects by moderating the expression of genes responsible for normal telomere length regulation. This is supported by our bipolar disorder sample, which shows that polygenic risk scores explain a higher proportion of the variance in telomere length amongst chronic lifetime lithium users (variance explained = 8.9%, *p* = 0.01), compared to non-users (*p* > 0.05). Consequently, this suggests that lithium may be catalysing the activity of endogenous mechanisms that promote telomere lengthening, whereby its efficacy eventually becomes limited by each individual’s inherent telomere maintenance capabilities. Our work indicates a potential use of polygenic risk scoring for the prediction of adult telomere length and consequently lithium’s anti-ageing efficacy.

## Introduction

‘Aging is not lost youth but a new stage of opportunity and strength’ [1].

Our population is ageing [2]; with increased longevity and decreased fertility rates, the median age of populations within more economically developed countries has risen from 28 in 1950 to 40 in 2010 [3]. Although longer life span has clear benefits, when it is associated with an increased proportion of the population suffering from age-related diseases, it can pose an economic burden [4]. Consequently, there has been an international effort to identify factors that can both increase longevity and delay the onset of morbidity [5].

One predictor of age-related disease, including coronary artery disease and obesity, as well as all-cause mortality and longevity, is telomere length [6]. Telomeres are stretches of TTAGGG nucleotide repeats at the ends of chromosomes [7]. They represent sacrificial DNA elements that protect vital coding DNA from being lost, as a result of the ‘end replication problem’; which is the loss of genetic material at the end of chromosomes (i.e. telomeres) each time a cell divides [8]. When a critical telomere length is reached, a cell loses the ability to divide [9]. This ultimately means that as we age, we are less able to replace old or damaged cells, and this can increase risk for age-related disease. Indeed, a direct relationship between telomere shortening and disease risk has been highlighted recently by Mendelian randomisation studies revealing that robust genetic predictors of telomere length also predict risk for coronary artery disease [10]. Therefore, telomere length represents both a biomarker for cellular age, and a potential anti-ageing drug target.

Psychiatric disorder patients exhibit high rates of comorbid age-related disease and frequently exhibit shorter telomere length relative to non-affected controls of a similar age [11]. Consequently, they represent a useful group in which to better understand the genetic and environmental contributions to shorter telomere length. Our recent work suggests a familial transmission of shorter telomere length, whereby even non-affected relatives of psychiatric disorder patients exhibit shorter telomeres compared to those with no family history [12]. Based on twin studies that reveal blood (leukocyte), telomere length is a highly heritable trait [13], and genome-wide association studies that reveal numerous loci as being involved in the regulation of its length [10], shared genetics could underlie this familial association. Indeed, we have previously shown that a genetic risk factor for shorter telomeres also confers risk for childhood-onset major depressive disorder [14]. However, in the more frequent cases of adult-onset psychiatric disorders, it appears that environmental factors (e.g. childhood stress) play a more important role in explaining shorter telomeres, than common genetic risk factors [15, 16]. Consequently, intervention strategies that focus on the environment may be particularly useful in preventing excessive telomere shortening amongst high-risk groups for age-related disorders, such as psychiatric disorder patients or those exposed to environmental trauma, and perhaps even more broadly for the general population.

In addition to stress, diet and medications can also affect rates of telomere shortening, suggesting that to an extent, we can actively moderate how we age [15–18]. This has led to the realisation that we may be able to pharmacologically reduce telomere shortening via the creation of anti-ageing (or anti-telomere shortening) medications. However, one of the pitfalls faced so far in targeting telomeres, pharmacologically, has been that excessive telomere length, and activity of the telomere-lengthening enzyme, telomerase, is associated with an increased risk of cancer [19]. Therefore, it’s likely that effective anti-ageing strategies would need to evoke subtle effects to telomeres across the life course, rather than rapid effects that may simultaneously increase cancer risk.

On a population scale, perhaps one of the most wide-reaching and effective ways to implement anti-ageing benefits across the life course would be by altering diet, and recent research suggests that even the water we drink may be important. Specifically, reports indicate that a higher level of lithium, naturally found in drinking water, is associated with fewer incidences of all-cause mortality, a reduced number of individuals committing suicide, increased longevity, and a reduced risk of neurodegenerative disease [20–23]. The anti-ageing benefits of lithium are not limited to humans either, with the effects being replicated in the worm *C. elegans*, and in the fly *Drosophila melanogaster*, where it extends lifespan [23–26].

In addition to being a metal naturally found in drinking water, lithium also has clinical applications, and is currently a first-line treatment for bipolar disorder (BD), where it acts as an effective mood-stabilizer [27]. BD patients are therefore a useful cohort to study the effects of lithium on anti-ageing mechanisms as these individuals are often taking relatively high, controlled doses for long periods of time. Indeed, we have shown in previous research that current lithium use is associated with longer telomere length amongst BD patients [12], and others have shown that lithium treatment duration amongst BD patients positively correlates with telomere length, specifically amongst chronic lifetime users [28, 29]. Lithium is further of interest, as its use is associated with longer telomeres, but with a decreased risk of cancer [30]. This accumulation of findings has sparked interest regarding the repositioning potential of lithium as an anti-ageing drug, and even the utility of lithium supplementation in drinking water (similarly to the use of fluoride for teeth), as a way to keep people healthier for longer [20, 31].

Due to lithium’s ability to affect a multitude of biological systems [32], more research is needed to understand its anti-ageing mechanism of action, and how generic its effects are in humans. For instance, research in *C. elegans* has shown a strong mediation of lithium’s anti-ageing effects by genetic factors [24], suggesting it may not be effective at preventing telomere shortening in a one-size-fits-all fashion across different genetic backgrounds. In humans, we know that variation in telomere length is moderated by a multitude of factors, such as oxidative stress and inflammation [33, 34], with perhaps the most pertinent factor being the activity of the telomerase enzyme, which adds TTAGGG repeats to telomere ends in dividing cells [35]. At least some of this inter-individual variation moderating telomere length is captured at the genetic level, for instance, single nucleotide polymorphisms (SNPs) within, or upstream of the telomerase genes represent the strongest predictors of leukocyte telomere length [10, 14]. In the case of lithium however, it’s currently unknown whether its telomere-lengthening effects work similarly for everyone (i.e. in a one-size-fits-all fashion), or whether variation in genes regulating baseline telomere length maintenance also contribute to variation in its anti-ageing benefits.

In this report, we determine: (i) the heritability of telomere length and confirm its genetic relationship to age-related disease and cancer, (ii) a polygenic risk score (PRS-TL) capable of predicting telomere length in an adult population, (iii) that chronic lithium use is associated with longer telomeres in an independent bipolar disorder sample, (iv) that genetic regulators of telomere length are affected in a *C. elegans* model of lithium-induced extended longevity and (v) that polygenic risk scores for telomere length explain a substantial proportion of inter-individual variability in telomere length amongst chronic lithium users, suggesting that lithium’s anti-ageing efficacy may be moderated by polygenic factors.

## Materials and methods

### LD score regression: heritability and genetic correlations

LD score regression via LD Hub (http://ldsc.broadinstitute.org/ldhub/) was used to estimate the SNP-chip heritability of telomere length, i.e. the proportion of variance in telomere length explained by common genetic differences [36]. To achieve this, we obtained genome-wide summary statistics directly from Codd and colleagues who performed the largest GWAS of telomere length to-date, using data from 37,684 individuals [10]. SNPs were merged to the recommended SNP list in LD Hub which excludes the major histocompatibility complex (MHC). In LD hub we further performed genetic correlations to test whether age-related phenotypes robustly associated with telomere length at the molecular level were mirrored at the genetic level. We limited our phenotypes to: (i) any cancer diagnosis (UK Biobank), (ii) body mass index (UK Biobank), (iii) coronary artery disease [37], (iv) low density lipoprotein [38] and high density lipoprotein [38].

### Bipolar association case-control study

Within this study we utilise 384 recurrent bipolar disorder patients recruited as part of the Bipolar Association Case-Control Study (BACCS) [39]. For full details on recruitment criteria, see S1 Supplementary information. Detailed phenotype data were also collected during the interview which included information on current lithium use, lifetime lithium use, duration of lithium treatment and lithium dose. Based on previous reports showing that lithium’s telomere-lengthening effects only correlate with duration of treatment amongst chronic lifetime users, (i.e. after several years of taking the drug) [28, 29], and because the treatment duration data was negatively skewed, we split our lifetime user group by the median treatment duration into two equally sized (and normally distributed) subgroups consisting of “short-term lifetime lithium users” ( < 4.5 years, *n* = 84), and “chronic lifetime lithium users” (4.5–30 years, *n* = 84). See Table 1 for sample characteristics. Access to molecular and clinical data related to BACCS is available upon request via a local access procedure, in accordance with the ethics agreement.

**Table 1:** Demographic data within the bipolar disorder sample

	Li-naive BD patients	Short-term lifetime Li users	Chronic lifetime Li users	Full sample
n	83	84	84	384
Age (mean, (SD))	44.48 (12.18)	44.06 (9.99)	52.14 (10.34)	48.42 (11.57)
Sex, n (% males)	22 (26.50)	30 (35.29)	35 (41.67)	126 (32.81)
BMI (mean, (SD))	26.20 (5.57)	28.24 (7.06)	28.08 (4.92)	27.42 (5.67)
Age of onset (mean, (SD))	21.08 (11.58)	20.69 (9.04)	22.31 (10.93)	21.63 (10.57)
Illness duration, years (mean, (SD))	24.26 (13.66)	22.63 (10.96)	30.26 (11.45)	21.21 (12.29)
Number of depressive episodes (mean, (SD))	11.60 (20.62)	11.96 (18.74)	14.08 (22.84)	12.22 (19.69)
Number of manic episodes (mean, (SD))	11.49 (22.70)	10.45 (18.85)	11.56 (19.94)	10.86 (19.30)
Number of mixed episodes (mean, (SD))	2.69 (12.25)	4.35 (16.03)	3.66 (13.29)	3.12 (12.72)
Current lithium use (n)	0	33	59	170
Ever taken other mood-stabilizers (n)	45	61	47	215
Ever taken antidepressants (n)	63	76	71	303
Ever taken antipsychotics (n)	49	64	63	250
Ever taken anxiolytics (n)	24	38	34	147

Information is based on available self-report data

### BACCS DNA extraction and preparation

25 mL of whole blood was taken from each participant at the time of interview and stored in EDTA blood tubes at −20 °C. Genomic DNA was then extracted using an inhouse protocol, previously described [40]. All DNA samples had 260/280 ratios of between 1.7 and 1.9, tested using the Nanodrop D1000 (Thermo Fisher Scientific, Massachusetts, United States), indicating good DNA purity.

### Telomere protocol

Relative telomere length (RTL) was quantified using a modified version of the quantitative Polymerase Chain Reaction (qPCR) protocol described by Cawthon and colleagues [41], as used by our lab previously [12, 16]. First, the protocol assayed the telomere variable repeat region (TTAGGG), and the cycle threshold (C_*t*_) required to reach a predetermined level of fluorescence: this correlated with the number of telomere repeats present in the individual samples. Second, and in parallel, a single-copy gene (albumin) was assayed in the same way, except the C_*t*_ now correlated with the number of copies of the genome in that individual DNA sample. Finally, a telomere-to-single-copy-gene ratio was used to determine RTL, where the number of telomere repeats in each sample was corrected for the total number of copies of that individual’s genome in the DNA sample being tested. See S2 for further details on the protocol and S3 and S4 in Supplementary information for the quality control procedures and results.

### BACCS genetic data

Genotype data was generated using Illumina HumanHap550 BeadChip (Illumina Inc., San Diego, CA, USA). Quality control was performed including the removal of SNPs with minor allele frequencies below 1%, and those not in Hardy-Weinberg equilibrium (*p* < 1 × 10^−5^), as described previously [42]. Multidimensional Scaling (MDS) in PLINK [43] was used to construct three population covariates (PCs), which were used in all analyses to correct for minor differences related to ancestry.

### Individualised polygenic risk scoring for telomere length

PRSice version 1.25 software [44] was used to determine the optimal *p*-value threshold (P_T_) where the polygenic risk for telomere length from the GWAS summary statistics [10] predicted telomere length in the BACCS cohort. To achieve this, our RTL data was initially adjusted for age, sex and BMI by taking the standardized residuals (z-scores); this phenotype was then modelled in PRSice with three ancestry PCs as covariates, for p-value thresholds from *p* = 0.001 to *p* = 0.5, increasing in 0.001 increments.

### Gene-enrichment analysis

MAGMA was applied to genome-wide summary statistics from the Codd et al. [10] GWAS, using the online tool FUMA [45]. MAGMA maps SNPs to genes in order to prioritise genes of functional relevance to a given trait. It generates a gene-wide statistic (and weighted *p*-value) from the GWAS results files, adjusting for gene size, single nucleotide polymorphism (SNP) density and linkage disequilibrium effects. We used a 10 kb 5′ and 3′ window around protein coding genes, as recommended by the authors, where genes surpassing genome-wide significance (*P* = 0.05/18879 = 2.648 × 10^−6^) were then investigated in datasets from a *C. elegans* model of lithium-induced longevity.

### eQTL analysis

We tested the effects of the most significant SNP associated with telomere length, rs10936599, on gene expression across multiple tissues using the online interface provided by the Genotype-Tissue Expression (GTEx) project [46].

### Lithium and C. elegans longevity microarray

Previous research in *C. elegans* assayed genome-wide expression changes associated with lithium-induced longevity [24]. Specifically, work by McColl and colleagues, revealed that a 10 mM dose of lithium increased the median lifespan of *C. elegans* by 46%. They subsequently assayed the genome-wide expression effects of a two-day 10 mM lithium treatment using a purpose-built *C. elegans* microarray (Genome Sequencing Center, University of Washington School of Medicine; Platform GPL5367 in GEO) to better understand the molecular mechanism conferring longevity. They found overlapping molecular effects of a two-day lithium treatment in C. elegans with effects observed in human cells treated in the same conditions. Microarray data is publically available from the Gene Expression Omnibus (GDS3140). OrthoList was used to identify *C. elegans* orthologs of human genes [47].

### Statistical analysis

#### Effects of lithium on telomere length

We tested the effect of lifetime lithium duration on RTL amongst short-term and chronic lifetime users separately, using a linear regression where RTL was the outcome, age, sex, BMI, current lithium use and three PCs were included as covariates, with lifetime lithium duration (weeks) as the independent variable. In the full bipolar disorder sample, we performed sensitivity analyses to test for the effects of (i) number of episodes (depressed/manic/mixed), (ii) other medications used, (iii) duration of illness and (iv) lithium dose, on RTL, with all models including age, sex, BMI and three PCs as covariates.

We additionally used data summary techniques to expand our current sample size and draw support from previous work on the effects of chronic lithium use on RTL. We included all primary human studies where the effects of lithium alone had been considered in the context of telomere length, by searching for “lithium” + “telomere” in PubMed (https://www.ncbi.nlm.nih.gov/pubmed). We conducted the data summarisation in R (https://www.r-project.org) using the package “metap” [48] and Stouffer’s “sumz” method [49], which allows for an estimation of z-scores and weighted average *p*-values in the absence of effect sizes. Weights of *p*-values from each study were defined as the square root of the sample size, as recommended by the authors.

#### Effect of PRS-TL on telomere length amongst lithium users and non-users

To compare the impact of PRS-TL in lithium-naive BD patients, short-term lifetime lithium users, and chronic lifetime lithium users, we performed a linear regression for each group of patients separately. RTL was selected as the outcome variable, with age, sex, BMI, current lithium use, lifetime lithium duration (weeks) and three PCs as covariates, with PRS-TL as the independent variable.

#### Effects of lithium on genetic regulators of telomere length in a model of extended longevity

Following MAGMA analyses, paired sample *t*-tests were used to compare whether implicated genes were affected in *C. elegans* following lithium treatment.

#### Multiple testing correction

For hypothesis-driven tests (effects of lithium on telomere length, genetic correlations on ageing traits) we considered *p* < 0.05 to be significant as these were replications of previous work. For all remaining analyses, we applied the Bonferroni method of multiple testing correction.

## Results

### Telomere length is polygenic and associated with risk for age-related disease and cancer

**Fig. 1:**
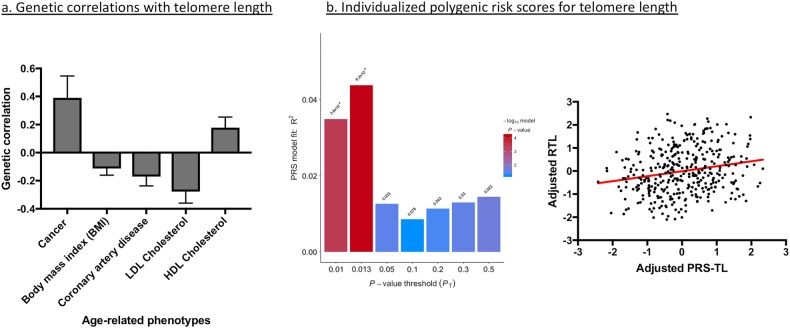
Genetic correlations with telomere length and individualised risk prediction. **a** Genetic correlations between single nucleotide polymorphisms predictive of increased telomere length and age-related phenotypes. **b** Left: Output from PRSice displaying a range of p-value thresholds (P_T_) tested, including the optimal P_T_ as shown in the tallest bar at threshold P_T_ = 0.013, which explained ~4.4% of the variance (*p* = 6.174 × 10^−5^). Right: A scatterplot showing the positive correlation between polygenic risk scores for telomere length (PRS-TL; adjusted for 3 PCs) and relative telomere length (RTL; adjusted for age, sex and BMI), Pearson (*r*) = 0.205, *p* ≤ 0.0001

LD score regression was applied to the largest telomere length GWAS to-date [10] in order to establish the proportion of variance in telomere length explained by common genetic differences. Results revealed a significant polygenic component to telomere length regulation, whereby there was a SNP heritability estimate of 7.29% (S.E. = 1.54). Polygenic risk for longer telomere length was associated with increased risk for cancer and HDL cholesterol, and decreased risk for coronary artery disease, high BMI and high levels of LDL cholesterol (all *p* ≤ 0.02) replicating epidemiological reports, Fig. 1. Polygenic risk scoring revealed that 3634 SNPs under the *p*-value threshold, P_T_ = 0.013 significantly predicted 4.382% of the variance in adjusted RTL (*p* = 6.174 × 10^−5^) in an independent sample of 384 bipolar disorder individuals, see Fig. 1. This effect remained significant after correcting for the 500 thresholds tested (adj. *p* = 0.03).

### Lithium use is associated with longer telomere length

**Fig. 2:**
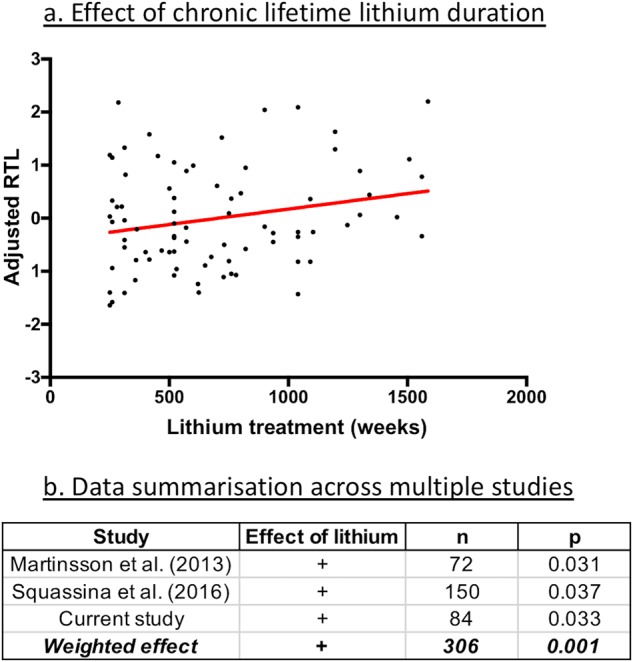
Lithium affects telomere length. **a** Scatterplot showing a positive association between lithium treatment duration and relative telomere length (RTL; adjusted for age, sex, BMI, PCs 1-3 and current lithium use) in chronic lifetime lithium users. **b** Data summarisation results using Stouffer’s sum of z method. Table includes previous studies assaying the effect of chronic lithium duration on RTL, the direction of effect observed (effect), sample size (n) and *p*-value (p), as well as a weighted effect combining results from all studies

Within our UK bipolar disorder sample (BACCS; see Table 1), short-term lifetime lithium duration did not predict telomere length (F(1, 75) = 0.47, *p* = 0.829, variance explained = 0.1%) as expected, but chronic lithium treatment duration did predict longer telomere length (F(1, 75) = 4.733, p = 0.033, variance explained = 6.3%), replicating previous findings [28, 29], Fig. 2. The effect of lithium treatment duration amongst chronic lifetime users was further validated using data summary methods (Stouffer’s z = 3.120 *p* = 9.061 × 10^−4^), Fig. 2. There were no effects of number of episodes, illness duration, other medications, or current lithium dose in the full BACC sample (see S5 Supplementary information). Amongst chronic lifetime lithium users, daily doses ranged from 120–1800 mg, but similarly to the full BACC sample, there were no effects of dose on RTL.

### Lithium targets genetic regulators of telomere length in a model of extended longevity

**Fig. 3:**
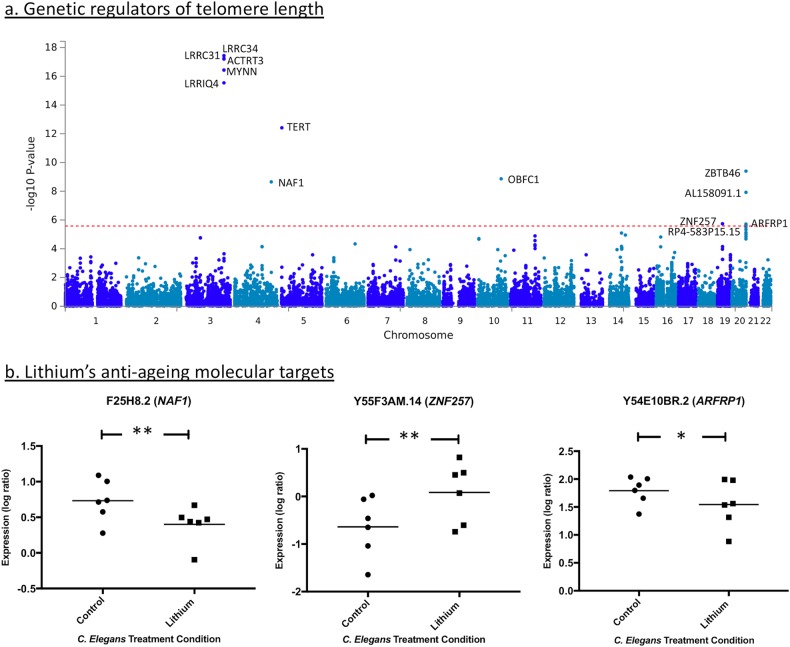
Genetic regulators of telomere length and effects of lithium. **a** Manhattan plot showing results from telomere length gene-enrichment analyses, indicating which genes are most important in affecting telomere length. The dashed line represents the threshold for genome-wide significance. **b** Three orthologs were assessed at the mRNA level in a *C. elegans* model of lithium-induced extended longevity, *p ≤ 0.05, **p ≤ 0.01

Our gene-enrichment analysis, using MAGMA and GWAS summary statistics from Codd and colleagues [10], revealed 13 genes significantly implicated in telomere length regulation, Fig. 3. eQTL analysis using GTex [46] confirmed an effect of the top telomere SNP rs10936599 on the expression of the most significantly enriched gene *LRRC34*, in transformed fibroblasts, adipose tissue, tibial nerve, heart, arteries, oesophagus and adrenal gland (all *p* < 3 × 10^−5^), whereby the risk allele for shorter telomere length (T-allele) was consistently associated with reduced *LRRC34* expression. There was no effect of rs10936599 on *TERC* expression levels. Of the 13 genes identified by our gene-enrichment analysis, three had orthologs in *C. elegans* that were also assayed in the McColl et al. study of lithium-induced extended longevity [24]. All three genes were differentially expressed upon lithium treatment in the model, including: *Y55F3AM.14* (human ortholog: *ZNF257*; t(5) = −3.884, *p* = 0.012), *F25H8.2* (human ortholog: *NAF1*; t(5) = 4.973, *p* = 0.004), and Y54E10BR.2 (human ortholog: ARFRP1; t(5) = 2.597, *p* = 0.048), Fig. 3. The effect on *Y55F3AM.14* and *F25H8.2* remained significant after correcting for three tests (adj. *p* < 0.05).

### Polygenic risk explains more inter-individual variability in telomere length amongst lithium users

**Fig. 4:**
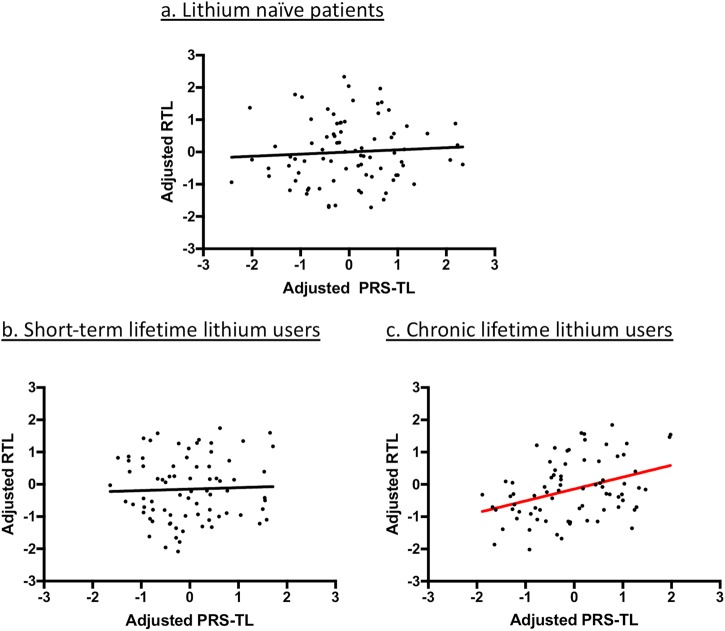
Lithium use and the effect of PRS-TL. Scatterplots showing the effects of polygenic risk scores for telomere length (PRS-TL; adjusted for PC’s 1-3), on relative telomere length (RTL; adjusted for age, sex, BMI and current lithium use), in **a** lithium-naive BD patients, **b** short-term lifetime lithium users and **c** chronic lifetime users. Significant correlations are indicated with a red line of best fit

To understand how PRS-TL behaves in lithium users and non-users, we tested its effect in lithium-naive BD patients, short-term lifetime lithium users and chronic lifetime lithium users, separately. The rationale for this was to understand if lithium works in a one-size-fits-all manner, or whether variation in PRS-TL explains inter-individual variation in telomere length amongst lithium users. In subsamples of just over 80 patients (Table 1), PRS-TL did not predict a significant amount of variance in RTL amongst BD patients who were naive to lithium (F(1, 72) = 0.250, *p* = 0.619, variance explained = 0.3%), nor in those who were short-term lifetime users (F(1, 75) = 0.85, *p* = 0.771, variance explained = 0.1%). In contrast, PRS-TL explained a relatively high proportion of the variance in RTL amongst chronic lifetime users (F(1, 75) = 6.802, *p* = 0.011, variance explained = 8.9%), see Fig. 4. The effect of PRS-TL in chronic lifetime users remained significant following multiple testing correction (adj. *p* = 0.033).

## Discussion

Telomere length represents a promising biomarker for age-related disease and a potential anti-ageing drug target. In this study we examined the genetic basis of telomere length regulation and explored the repositioning potential of lithium as an anti-ageing medication. First, our study revealed that telomere length is a polygenic trait with SNP heritability estimates of 7.29%. Using polygenic risk scoring we identified a genetic score which explained 4.4% of the variance in telomere length in an independent sample, which is an improvement to the previously reported genetic risk score consisting of only genome-wide significant SNPs that explained just over 1% [10]. These findings further support twin research suggesting telomere length is a highly heritable trait, but our work also suggests that a significant amount of variation remains missing (up to 74%), which may indicate that even larger sample sizes and more powerful GWAS are required, or that rare variants, gene-environment interactions or epigenetic modifications also add significantly to twin heritability estimates [13]. Genetic correlations corroborate previous reports that indicate a higher risk for cancer amongst individuals with very long telomeres [19]. In terms of age-related disease phenotypes, we found genetic risk for longer telomeres was associated with higher levels of high density lipoprotein (the ‘good cholesterol’) and reduced levels of low density lipoprotein (the ‘bad cholesterol’), alongside a reduced risk for coronary artery disease and high body mass index. This supports a multitude of studies that indicate a strong relationship between telomere length and age-related risk for coronary artery disease [10, 50, 51].

To better understand what genes are functionally important in regulating telomere length, we performed gene-level enrichment analysis on GWAS summary data. We found that the top five genes associated with telomere length were all clustered around the same genomic location on chromosome 3. These adjacent genes fall upstream of the telomerase gene *TERC*, and consequently it’s possible that a range of SNPs exerting long range cis-regulatory effects on *TERC* are inflating signal in this genomic area. To gain a better grasp on what SNPs in this area affect which genes, we performed expression quantitative trait loci (eQTL) analysis on the most significant SNP associated with telomere length (rs10936599). This analysis did not reveal any effect of rs10936599 on *TERC* but did reveal an effect of the SNP on the most significantly enriched gene, leucine rich repeat containing 34 (*LRRC34*), across multiple tissue types, whereby the T-allele (associated with shorter telomere length) was consistently associated with reduced expression. Although the exact role of *LRRC34* is unclear, it is predicted to act as a ribonuclease inhibitor [52]. As a key component of telomerase’s mechanism is the temporary incorporation of a non-coding RNA template to the lagging strand of DNA at our chromosome ends, it’s possible that ribonuclease inhibitors help to preserve the RNA primer pivotal to telomere restoration. Consequently, its plausible that the LLR genes proximal to *TERC* are independently important in the regulation of telomere length, however further functional studies (e.g. CRISPR) will be needed to gain a definite understanding of how SNPs in these regions exert their effects. Other genes identified from our analyses included previously implicated regulators of telomere length (*TERT*, *NAF1*, *OBFC1*, *ZBTB46*, *ZNF257*) and some novel genes (*AL158091.1*, *RP4-583P15.15*), which will require further work to better understand their function [10].

Next, we confirmed that chronic lifetime lithium use is associated with longer telomere length in an independent sample of 384 BD patients, and in an expanded sample [12, 28, 29]. This supports epidemiological data which has shown that lithium in our water supply has beneficial effects on health and longevity and suggests that lithium’s effect on telomere length may be one mechanism by which it confers its anti-ageing properties [20, 23]. To corroborate this theory, we tested whether lithium affects the expression of genes responsible for telomere length maintenance (identified from our gene-enrichment analyses) in a relevant model system that recapitulates the drug’s anti-ageing effects. We found that 3 out of the 13 genes identified from the gene-enrichment analysis had an assayed ortholog in a *C. elegans* model of lithium-induced extended longevity, where we found that lithium had an effect on all three genes. This subsequently supports the notion that genes responsible for normal telomere length regulation may play a role in mediating lithium’s anti-ageing mode of action.

Finally, we used PRS-TL to better understand whether SNPs involved in telomere maintenance contribute to inter-individual variation amongst lithium users, or whether lithium’s telomere-lengthening effects work the same for everyone. Our results revealed variation in telomere length amongst lithium users, with a substantial proportion being explained by PRS-TL. In fact, far greater variance in telomere length was explained by PRS-TL in chronic lifetime lithium users (8.9%) relative to lithium-naive BD patients (0.3%) and short-term lifetime users (0.1%). This disparity suggests that lithium is not simply extending telomere length in a one-size-fits-all fashion, with residual baseline differences between individuals remaining, rather that lithium is increasing the penetrance of genetic differences in telomere length. In light of the results from the *C. elegans* model, it further suggests that lithium may be catalysing the activity of endogenous mechanisms responsible for telomere lengthening via its effects on gene transcription, whereby it eventually approaches a plateau and its efficacy becomes limited by each individual’s inherent telomere maintenance capabilities, as captured using PRS-TL.

In sum, our findings have several potential implications. Our polygenic risk scoring result suggests that common genetic differences can predict over 4% of the variance in adult telomere length. This supports the possibility that PRS-TL may eventually represent a useful way of predicting those at risk for age-related disease (or cancer), though this will need to be verified in independent studies. It also adds support for further larger telomere GWAS to be performed in order to observe whether we can increase the predictive power of our PRS. Our comparative genomics work revealed that lithium can moderate the expression of genes governing telomere length, and this might be one mechanism via which it extends telomeres amongst bipolar disorder patients. Consequently, lithium may have repositioning potential for its anti-ageing effects in susceptible individuals. For instance, studies have shown that childhood maltreatment can shorten telomeres, which is a possible mechanism via which these individuals are also at higher risk for age-related disease [16]. Therefore, if telomere length was confirmed to be shorter amongst a maltreated individual, lithium might be a treatment option to prevent further premature ageing. Our results also suggest that lithium would likely be most effective if that individual also has a genetic predisposition to having longer telomeres in the first place (captured by PRS-TL). Thus, a combination of information on an individual’s exposure to telomere-shortening environmental risk factors (e.g. by a childhood trauma questionnaire), confirmation of shorter telomere length via molecular probing (e.g. qPCR), and quantification of genetic risk for telomere length (e.g. PRS-TL), could be useful in identifying individuals who will need, and respond best, to the anti-ageing benefits of lithium.

Although the work in BD patients reported here represents a microcosm of how lithium supplementation may act on a population level, the results are encouraging, and adds support to epidemiological data which finds associations between higher lithium levels in water supplies and lower risk for age-related disease [20, 23]. We now need to study how lithium acts in a far larger, non-clinical population setting and confirm that the genetic factors restricting lithium’s benefits identified here, replicate in other contexts. Furthermore, although, chronicity of treatment seems to be more important than lithium dose based on our analyses, we still need to consider how comparable low lithium levels are to the high clinical levels used to treat BD. Moreover, when considering doses of lithium for repurposing we should be mindful that high doses can be toxic, and are related to thyroid dysfunction, kidney injury, blood dyscrasias, and polydipsia, all of which can shorten lifespan [53]. Therefore, careful consideration of upper dose limits and further refinement of the optimal therapeutic range of lithium for anti-ageing purposes will need to be considered in the future.

There are a number of other limitations in this report that should also be acknowledged. First, the study makes a number of inferences about the effects of lithium based on associations and the use of genetic predictors, but ultimately prospective longitudinal data and functional studies are required to confirm our findings and to better understand how lithium mediates its telomere-lengthening effects in the context of different genetic backgrounds. Second, our BD sample size is relatively small and our study utilises samples from a severe clinical population on high doses of lithium, and therefore the results may not be representative of the wider unaffected population. Third, analysis using the *C. elegans* model may not reflect what is observed in humans. For instance, the 10 mM dose applied is ten times that which is found in the serum of BD patients and would be considered toxic for humans [54]; although the authors found that this dose was not toxic in their model, and it is generally accepted that smaller organisms require higher doses of drugs due to their faster metabolisms [55]. Future longitudinal studies assessing the effects of lithium in the context of telomere length and age-related disease risk will be best placed to confirm which gene transcripts are important in mediating lithium’s telomere-lengthening effects. Fourth, although our polygenic predictor captures a significant amount of the variance in adult telomere length, the effect is still small, and may not be clinically useful in predicting age-related disease risk, or it may only be valuable when combined with disease-specific environmental risk factors [56]. Despite these limitations, our results extend previous work on the genetics of telomere length and confirms the potential utility of lithium as an anti-ageing compound, though we acknowledge that lithium’s effects may be limited by the same polygenic factors responsible for baseline telomere length maintenance.

## Supplementary information

Supplementary information
