# Supplementary material for: The polygenic nature of telomere length and the anti-ageing properties of lithium
Source: Neuropsychopharmacology. 2018 Dec 18;44(4):757–65. doi: 10.1038/s41386-018-0289-0 (PMC6372618; doi:10.1038/s41386-018-0289-0)
Supplement: Supplementary file 1 — Supplementary information [file 41386_2018_289_MOESM1_ESM.docx]

The polygenic nature of telomere length and the anti-ageing properties of lithium

Fiona Coutts^1#^, Alish B. Palmos^1#^, Rodrigo R. R. Duarte^1,2^, Simone de Jong^1^, Cathryn M. Lewis^1,2^, Danai Dima^3,4^, Timothy R. Powell^1*^

**Supplementary information**

**S1: BACC Study Recruitment Criteria**

**S2: Telomere Protocol**

**S3: Telomere Quality Control Criteria**

**S4: Telomere Quality Control Results**

**S5: The effects of confounders on telomere length**

**S1: BACC Study Recruitment Criteria**

All participants had been diagnosed with Bipolar I or Bipolar II disorder as defined by the Diagnostic and Statistical Manual 4th edition operational criteria (DSM-IV). The exclusion criteria were: i) if a first degree relative met criteria for schizophrenia, ii) if the BD patient presented psychotic symptoms with no link to mood, iii) if intravenous drug use or drug dependency had ever occurred, iv) if mania/depression ever occurred solely due to alcohol or substance use or medical illness, v) if the patient was related to an individual already in the study. BD patients were identified from psychiatric clinics, hospitals, primary care physicians, patient support groups, and from volunteers responding to media advertisements. All participants were interviewed in person using the Schedules for Clinical Assessment in Neuropsychiatry (SCAN). Blood was taken from each participant for DNA extraction and subsequent genetic analyses. All patients were euthymic (not in a current mood episode) at the time of recruitment. Written informed consent was obtained from all participants and the study was approved by the Joint South London and Maudsley Ethics Committee.

**S2: Telomere Protocol**

Per 96 samples assayed, two 384-well plates were prepared in parallel; one to assay the telomere repeat region, and one to assay the albumin gene. A seven-point standard curve of doubling concentrations (0.47 ng, 0.94 ng, 1.88 ng, 3.75 ng, 7.5 ng, 15 ng, 30 ng) was set up by serial dilution to allow for absolute quantification of the amplified DNA, and to determine and control for the efficiency of the qPCR reactions. Four negative controls containing RNase-free water instead of DNA were used to test for DNA contamination, and five positive controls containing leukocyte DNA from five separate individuals were included on every plate to confirm successful PCR amplification. For all samples, the seven-point standard curve and the positive and negative controls, three technical replicates were used. The same sample well positions were used for both the telomere and albumin plates.

Each telomere reaction was made up to 15 μL per well, containing 10 μL SYBR green Primer Design Mastermix (2x) (Primer Design, Southampton, UK), 5 μL of RNase-free water, 1000 nM of the telomere forward primer (5’-ACACTAAGGTTTGGGTTTGGGTTTGGGTTTGGGTTAGTGT-3’), 800 nM of the telomere reverse primer (5’-TGTTAGGTATCCCTATCCCTATCCCTATCCCTATCCCTAACA-3’), and 12 ng of DNA. The thermocycling reaction (performed on the Quantstudio 7 Flex Real-Time PCR System) was set up as shown in **Figure 1a**. The albumin qPCRs were set up in the same way but instead using 765 nM of the albumin forward primer (5’-CGGCGGCGGGCGGCGCGGGCTGGGCGGAAATGCTGCACAGAATCCTT-3’) and 930 nM of the albumin reverse primer (5’-GCCCGGCCCGCCGCGCCCGTCCCGCCGG AAAAGCATGGTCGCCTGTT-3’), and using the thermocycling conditions seen in **Figure 1b**.

95.0

0.15

**Stage 4**

60.0

0.15

95.0

0.15

62.0

0.10

73.0

0.15

**Stage 3**

94.0

0.15

49.0

0.15

94.0

0.15

95.0

15.00

**Stage 1**

**Stage 2**

Repeats: 2

Repeats: 25

95.0

0.15

**Stage 4**

60.0

0.15

95.0

0.15

62.0

0.10

88.0

0.15

**Stage 3**

94.0

0.15

49.0

0.15

94.0

0.15

95.0

15.00

**Stage 1**

**Stage 2**

Repeats: 2

Repeats: 33

**a)**

**b)**

**Figure 1 – Thermocycling conditions**

a) The telomere reaction, and b) the albumin reaction. The upper values represent temperature (°C) and the lower values represent time (minutes.seconds). Data was collected in the last step of Stage 3 for both reactions. Stage 4 represents the dissociation curve.

**S3: Telomere Quality Control Criteria**

Initial analysis of the standard curve, melt curve and controls was performed using QuantStudio™ Real-Time PCR Software. Subsequently, any replicates with C*_t_* standard deviations > 0.5 from the other two were excluded from further analysis. Samples that still had a mean C*_t_* standard deviation of greater than 0.5 after exclusion of one replicate were removed from downstream analysis. Remaining mean C*_t_* values were then related to the standard curve in order to obtain absolute quantities of DNA for each sample (C*_q_*). The RTL values were then calculated for each sample by dividing the mean C*_q_* for the telomere reaction by the mean C*_q_* for the albumin reaction. The RTL values were log-transformed for parametric use and adjusted for plate batch by taking the standardized residuals to control for the effects of minor inter-plate variability. Outliers were defined as those that were ±2 standard deviations from the mean.

**S4: Telomere Quality Control Results**

All ten 384-well plates and all samples passed quality control criteria. All telomere and albumin standard curves had an R^2^>0.985, allowing for the accurate use of a standard curve. The mean PCR efficiency was 99.4% for the telomere reaction and 87.8% for the albumin reaction, which is in keeping with previous reports. Differences between the efficiencies for the two primer sets are corrected for via the use of a standard curve. The melt curves showed no evidence of non-specific amplification or primer dimer, confirming that the amplification was specific to the required region (**Figure 2**). The negative controls showed no amplification, confirming that there was no nucleic acid contamination.


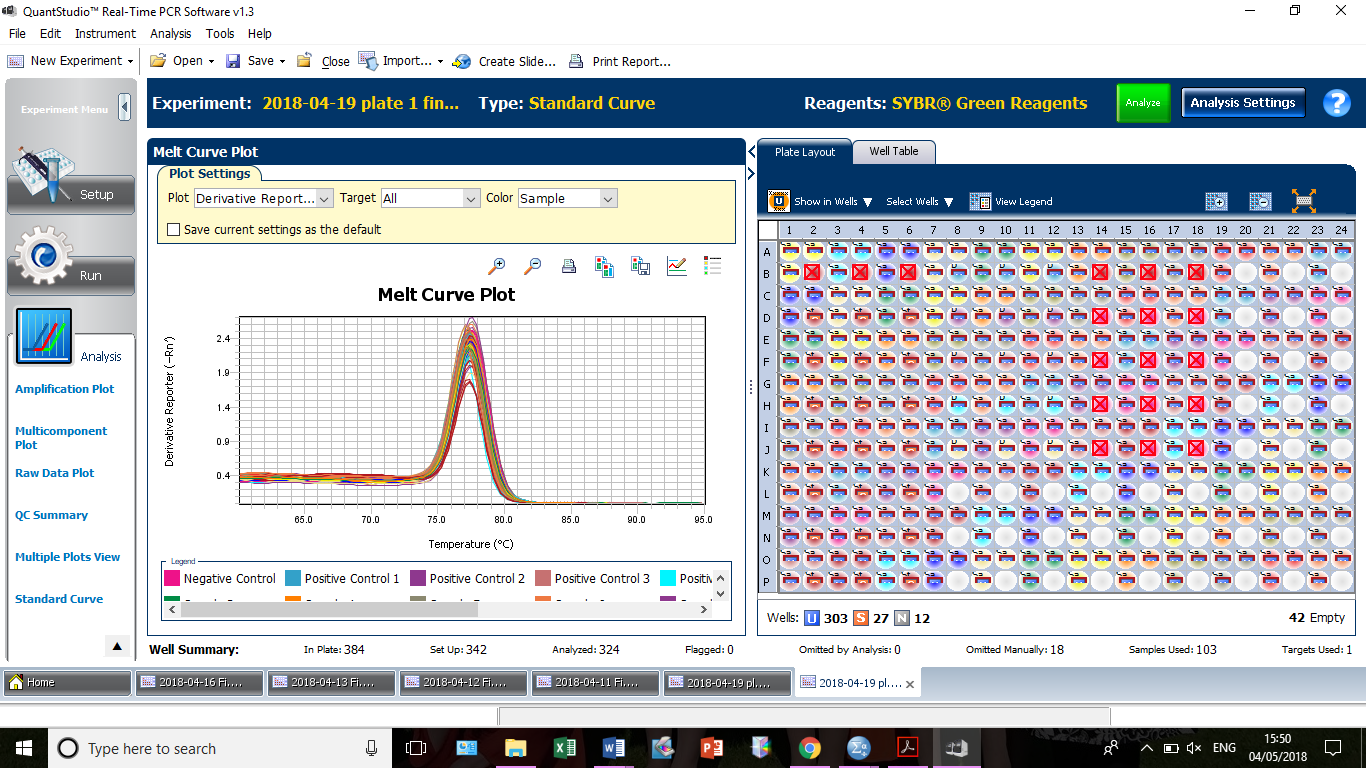


**a)**

**b)**


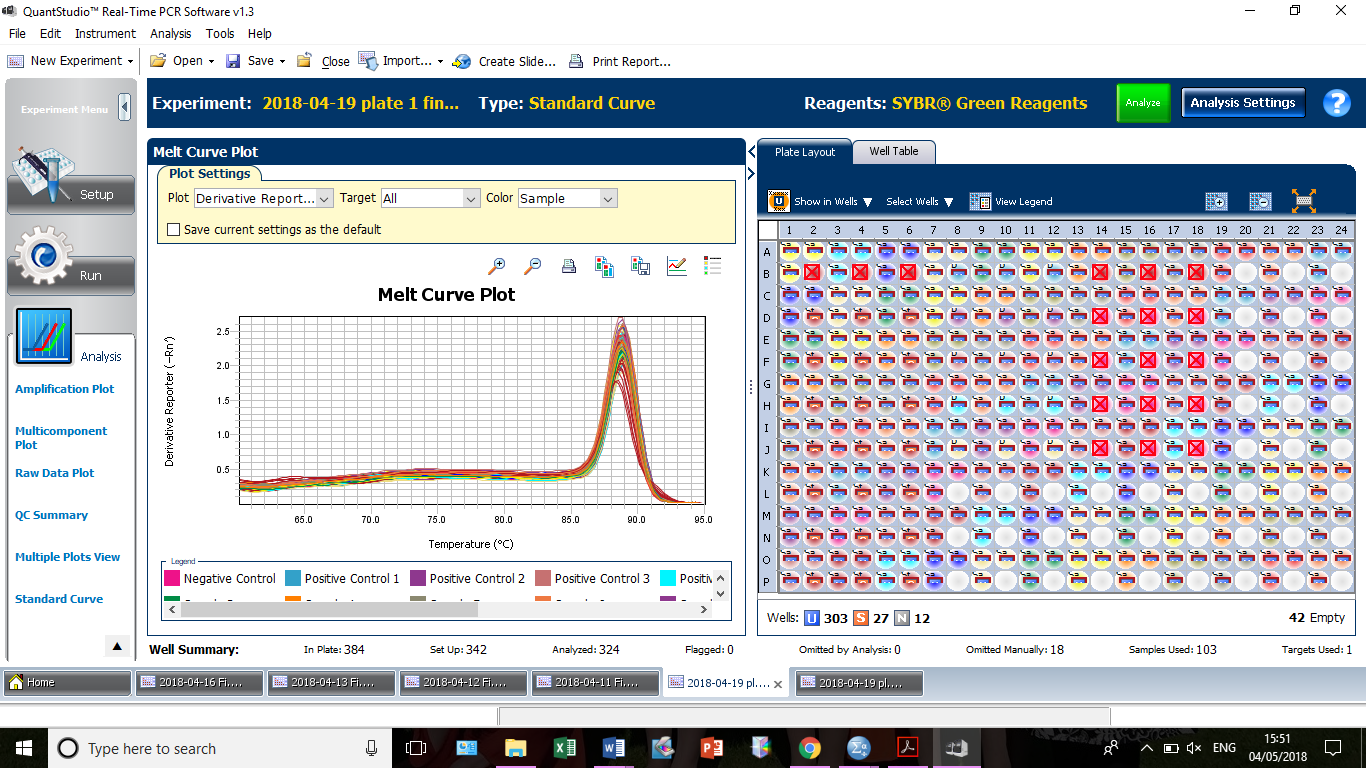


**Figure 2 – Example melt curve plots**

**a)** The telomere reaction, and **b)** the albumin reaction

**S5: The effects of confounders on telomere length**

In our total sample we tested for the effects of potential confounders on RTL, with all models including age, sex, BMI and three PCs as covariates. RTL was not significantly affected by: number of manic episodes (F(1, 337) = 0.562, p = 0.454, variance explained = 0.2%); number of depressed episodes (F(1, 330) = 1.113, p = 0.292, variance explained = 0.3%); number of mixed episodes (F(1, 342) = 0.352, p = 0.554, variance explained = 0.1%); duration of illness (F(1, 292) = 0.344, p = 0.558, variance explained = 0.1%); lifetime use of other mood-stabilizers (F(1, 350) = 0.609, p = 0.436, variance explained = 0.2%); lifetime use of antidepressants (F(1, 337) = 0.723, p = 0.396, variance explained = 0.2%); lifetime use of antipsychotics (F(1, 336) = 0.445, p = 0.505, variance explained = 0.1%); lifetime use of anxiolytics (F(1, 336) = 1.667, p = 0.198, variance explained = 0.5%); or current lithium dose (F(1, 144) = 1.524, p = 0.219, variance explained = 1%).
